# Supplementary material for: Engineering synthetic signaling receptors to enable erythropoietin-free erythropoiesis
Source: Nat Commun. 2025 Jan 29;16:1140. doi: 10.1038/s41467-025-56239-5 (PMC11779867; doi:10.1038/s41467-025-56239-5)
Supplement: Supplementary file 1 — Supplementary Information [file 41467_2025_56239_MOESM1_ESM.pdf]

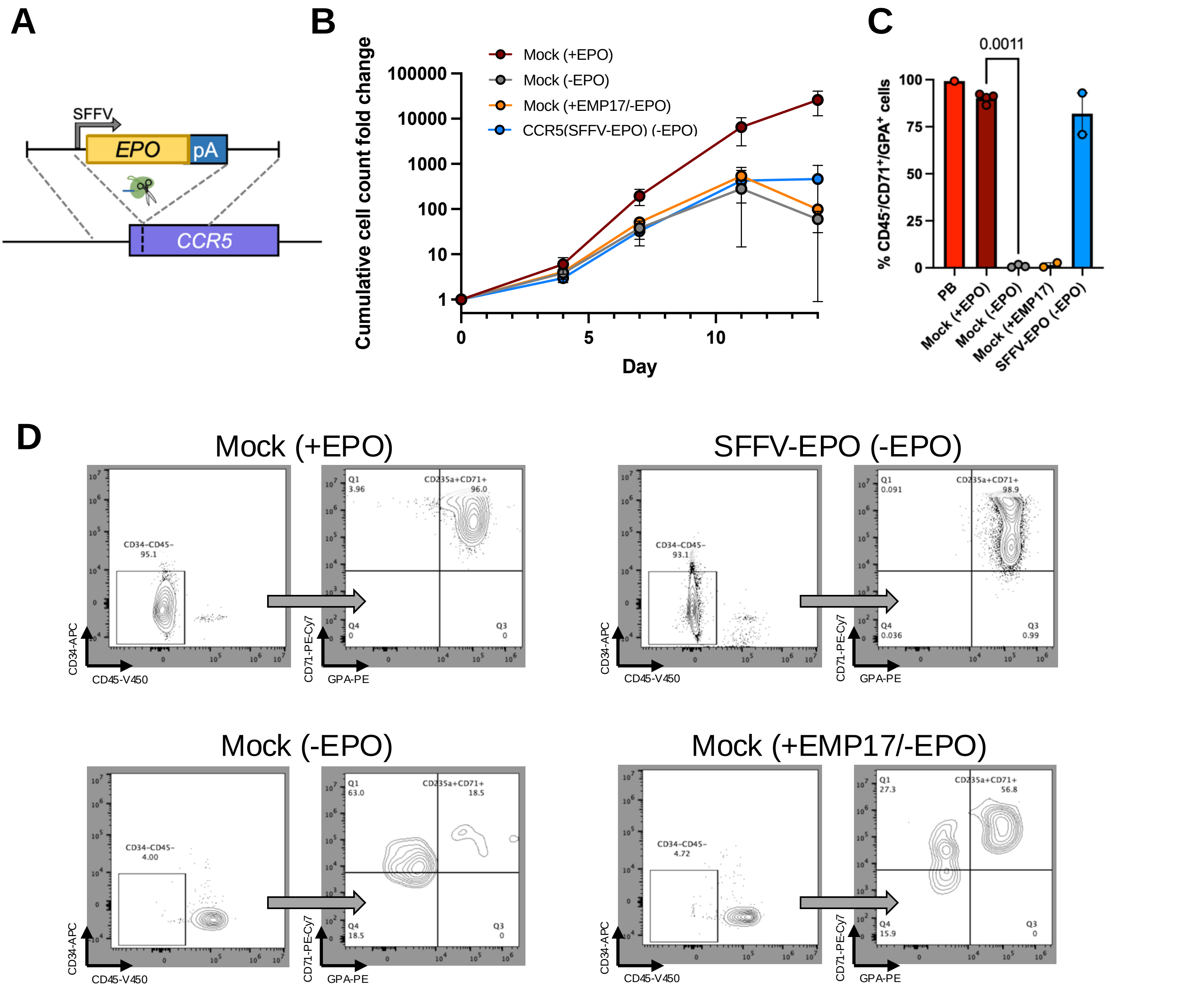

Supplementary Figure 1: Alternative strategies to mediate EPO-free erythropoiesis.

A: Schematic of genome editing strategy to introduce an SFFV promoter driving expression of an *EPO* transgene from the *CCR5* safe harbor site.

B: Cumulative cell count fold change over the course of erythroid differentiation. Bars represent median +/-SEM. N=5 biological replicates for Mock condition +EPO/-EMP17; N=3 biological replicates for Mock condition -EPO/-EMP17; N=2 biological replicates for Mock condition -EPO/+EMP17 and *CCR5*(SFFV-EPO) condition.

C: Percentage of HSPCs that acquired erythroid markers (CD34-APC^{-}/CD45-V450^{-}/CD71-PE-Cy7^{+}/GPA-PE^{+}) +/-BB normalized to unedited cells +EPO at d14 of differentiation. PB represents peripheral blood isolated from a healthy human donor. Bars represent median +/-SEM; displayed p-value was generated by 2-way ANOVA for multiple comparisons.

D: Representative flow cytometry staining and gating scheme for HSPCs at d14 of erythroid differentiation. Arrows indicate that only gated cells are displayed on the subsequent plot.

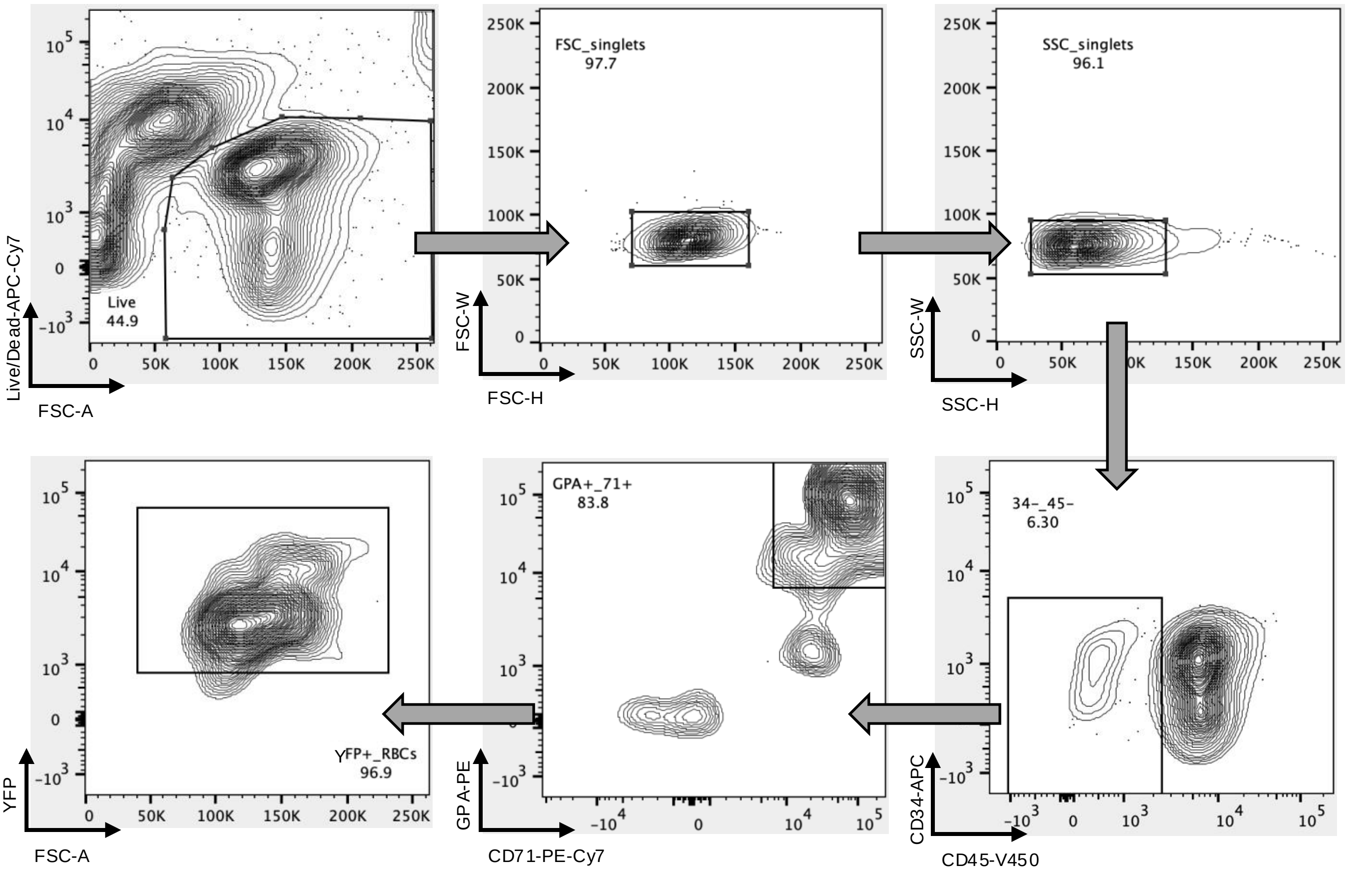

Supplementary Figure 2: Representative flow cytometry staining and gating scheme for erythroid differentiation analysis.

HSPCs were edited with synEPOR 1.5, subjected to *ex vivo* HSPC-to-erythroid cell differentiation in absence of EPO but presence of BB, and analyzed at day 14. Arrows indicate that only gated cells are displayed on the subsequent plot.

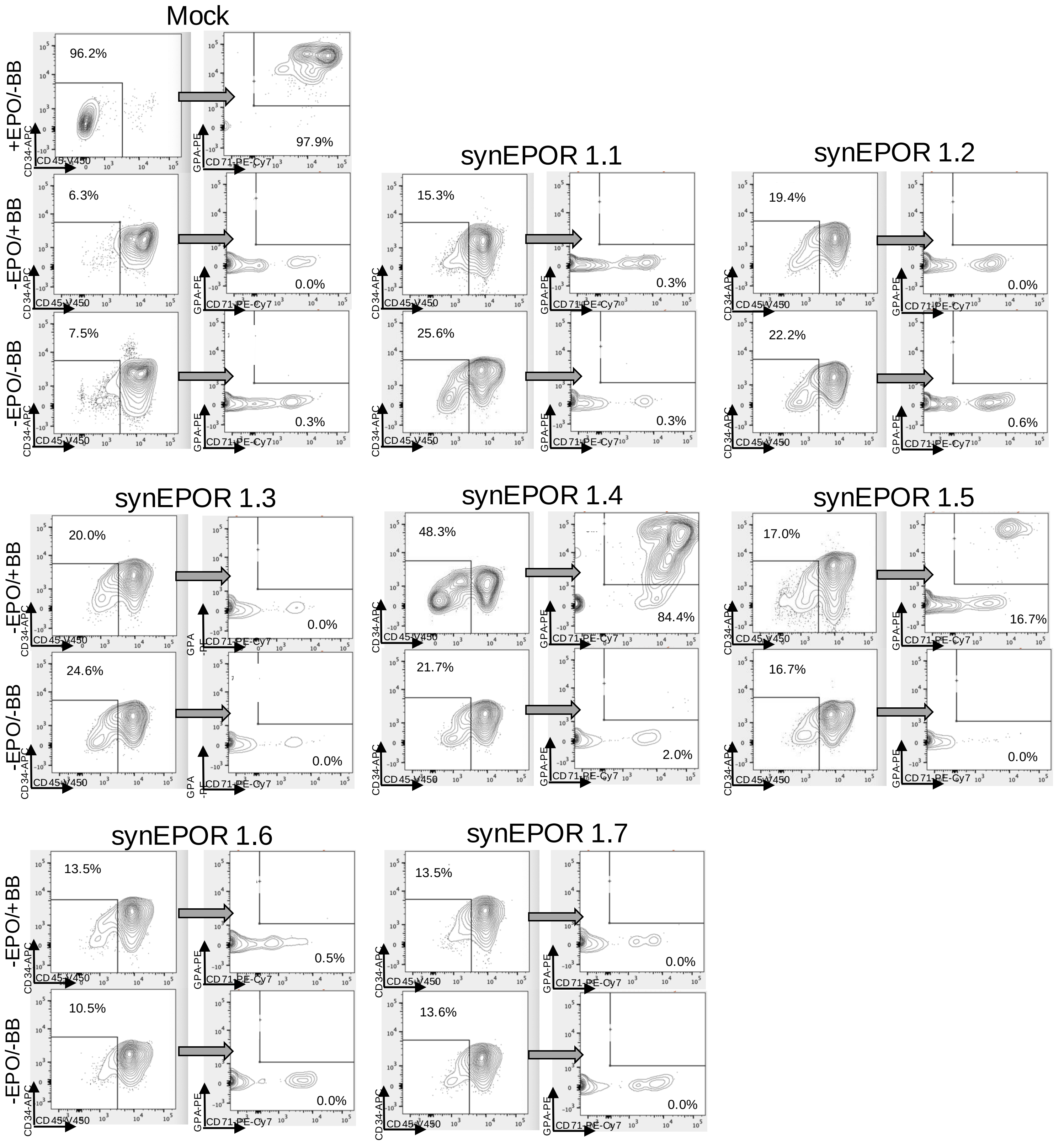

Supplementary Figure 3: Flow cytometry analysis of first-generation synEPORs.
Representative flow cytometry staining and gating scheme for first-generation synEPOR-edited HSPCs at d14 of erythroid differentiation. Arrows indicate that only gated cells are displayed on the subsequent plot.

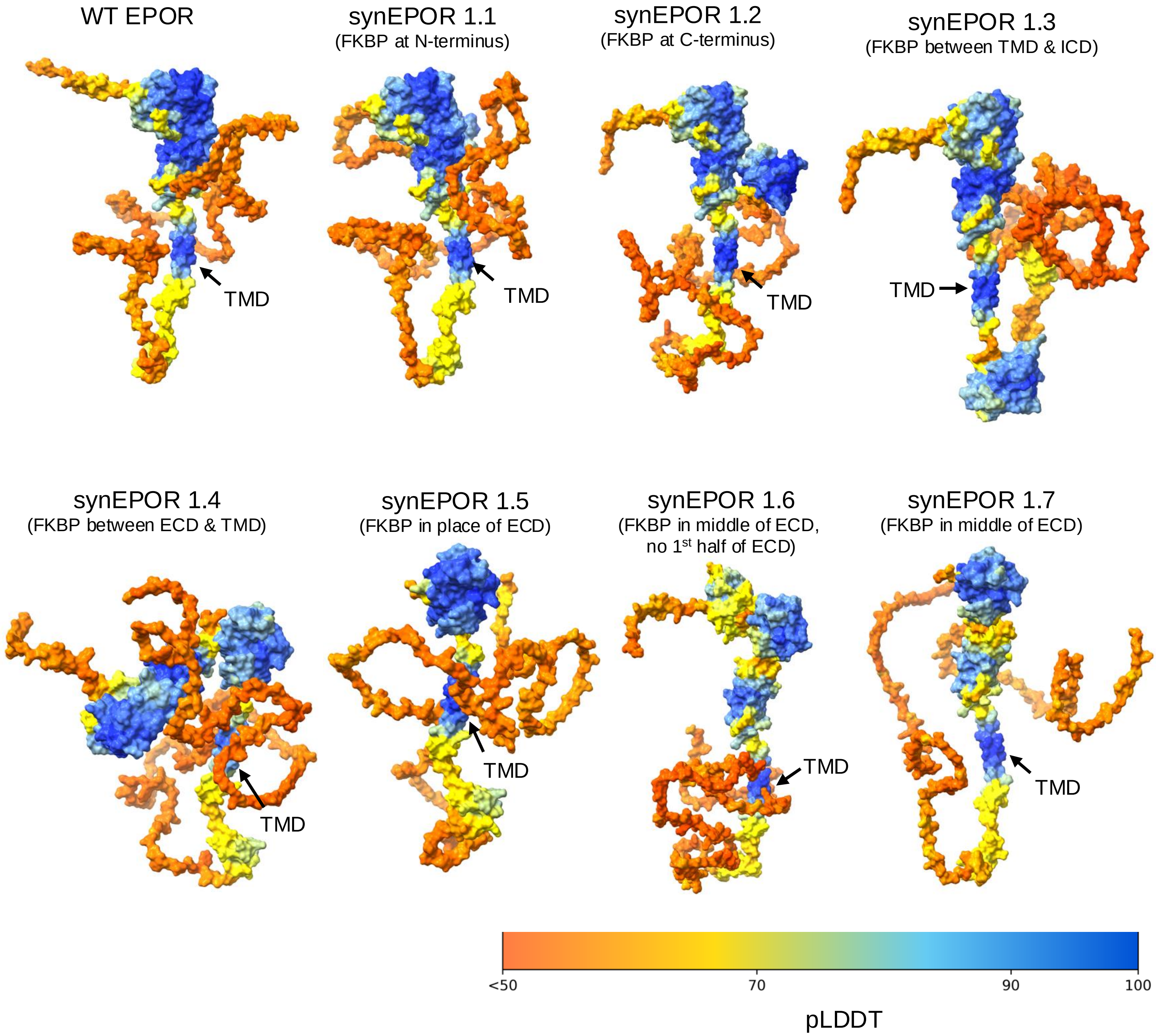

Supplementary Figure 4: *In silico* structure prediction of first-generation synEPORs.
AlphaFold2-based structure prediction of wild-type (WT) EPOR and all candidate synEPORs. As a measure of structure confidence, predicted distance difference test (pLDDT) is displayed according to the color bar. Abbreviations as follows: ECD = extracellular domain; TMD = transmembrane domain; and ICD = intracellular domain. TMD labeled with an arrow as a reference point.

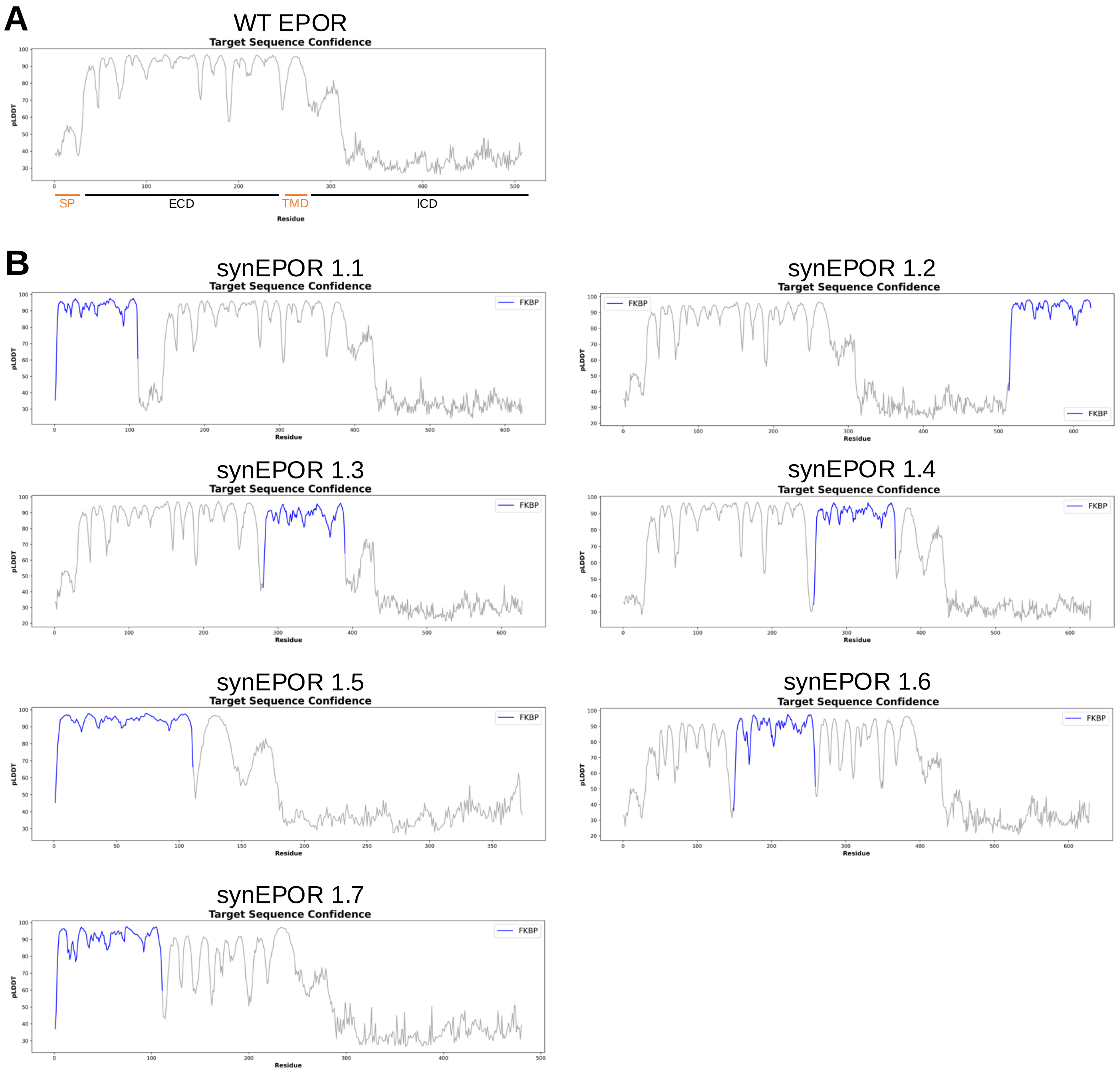

Supplementary Figure 5: *In silico* structure prediction of first-generation synEPORs.
A: Plot of pLDDT of AlphaFold2-predicted structure for WT EPOR shown in Supplementary Figure 4. Annotations are as follows: SP = signal peptide; ECD = extracellular domain; TMD = transmembrane domain; and ICD = intracellular domain.
B: Plots of pLDDT of predicted structures for all candidate synEPORs shown in Supplementary Figure 4A. FKBP domain is highlighted in blue.

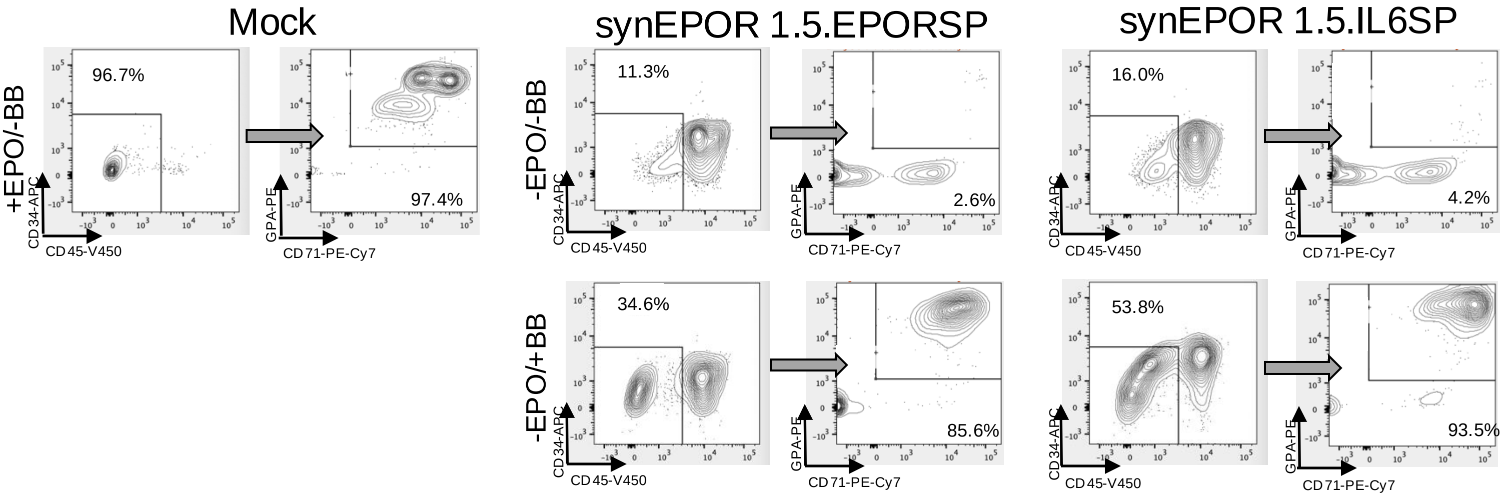

Supplementary Figure 6: Flow cytometry analysis of second-generation synEPORs.
Representative flow cytometry staining and gating scheme for second-generation synEPOR-edited HSPCs at d14 of erythroid differentiation. Arrows indicate that only gated cells are displayed on the subsequent plot.

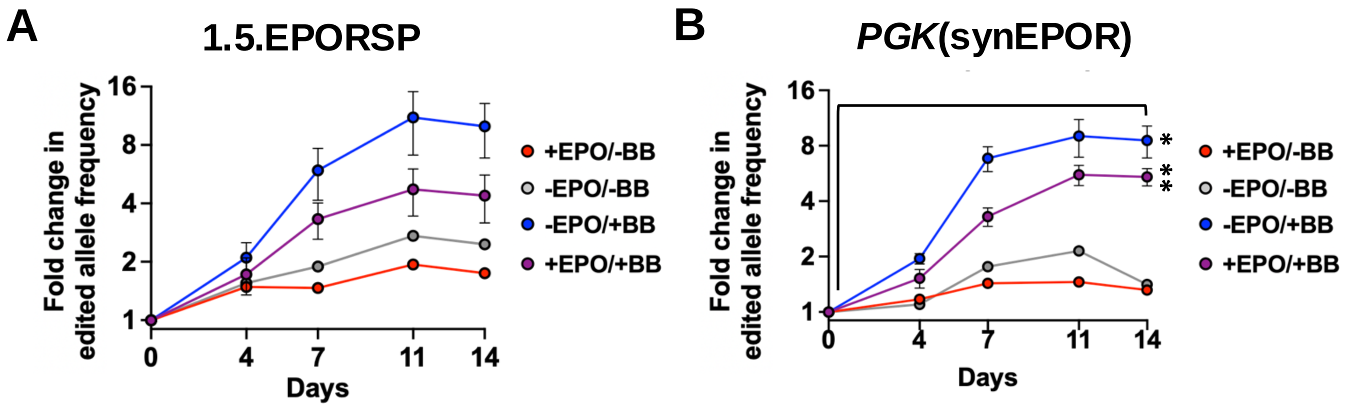

Supplementary Figure 7: Enrichment of edited cells over course of erythroid differentiation.

A: Fold change of edited allele frequencies following editing with 1.5EPORSP measured by ddPCR over the course of differentiation +/-EPO and +/-BB. Bars represent mean +/-SEM. N=3 biological replicates for all conditions.

B: Fold change of edited allele frequencies following editing with *PGK*(synEPOR) measured by ddPCR over the course of differentiation +/-EPO and +/-BB. Bars represent mean +/-SEM; *: p=0.00421 and **: p=0.000019 comparing +BB/-EPO treatment at d0 vs. d14 by unpaired two-tailed t-test across distinct samples. N=6 biological replicates for -BB/+EPO and +BB/+EPO conditions; N=4 biological replicates for +BB/-EPO condition; and N=2 biological replicates for -BB/-EPO condition.

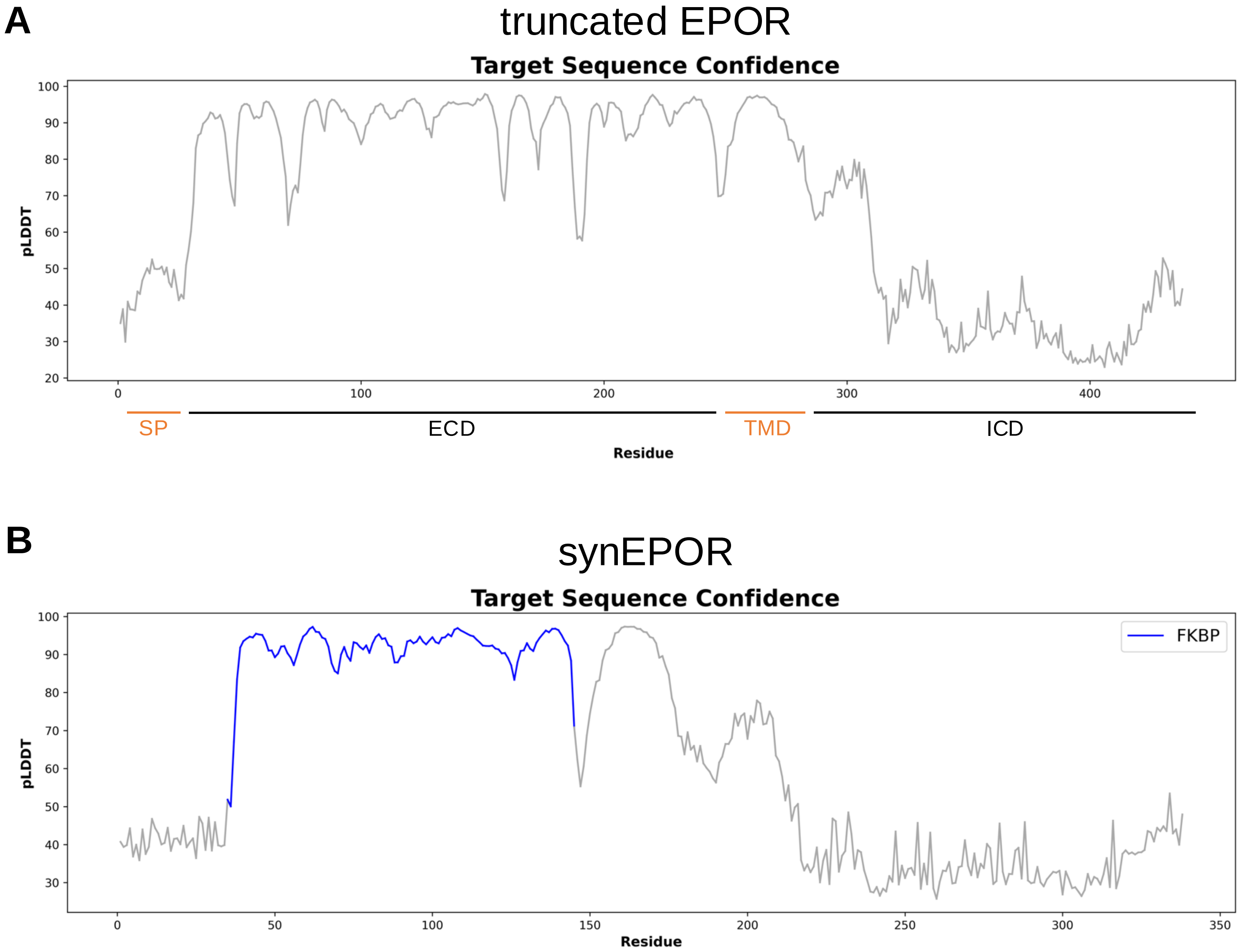

Supplementary Figure 8: *In silico* structure prediction of final synEPOR design.
A: Plot of pLDDT of AlphaFold2-predicted structure for truncated EPOR shown in Fig. 2F. Annotations are as follows: SP = signal peptide; ECD = extracellular domain; TMD = transmembrane domain; and ICD = intracellular domain.
B: Plot of pLDDT of predicted structure for the optimized synEPOR shown in Fig. 2F. FKBP domain is highlighted in blue.

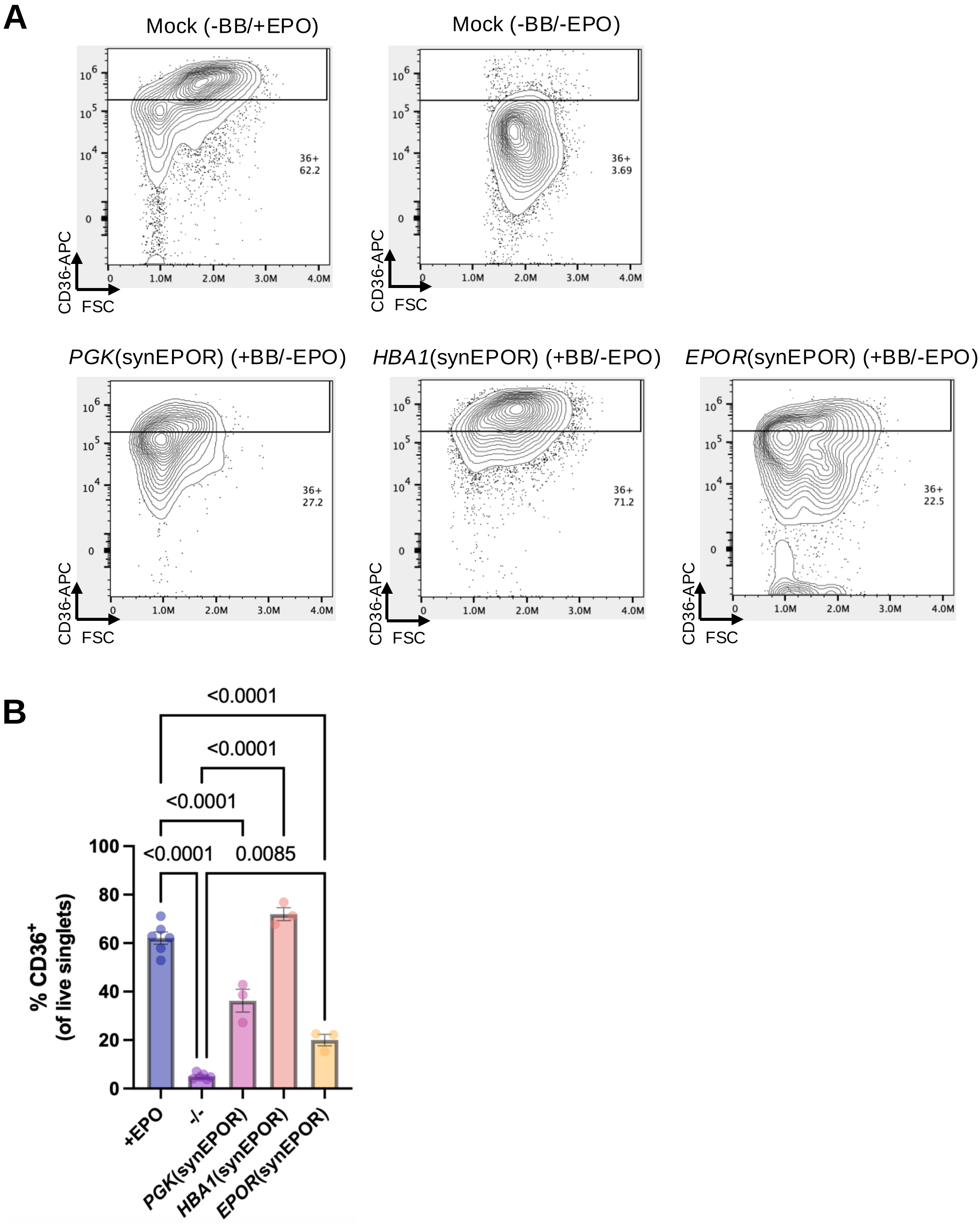

Supplementary Figure 9: Deeper characterization of synEPOR-edited HSPCs using flow cytometry.

A: Representative flow cytometry staining and gating scheme for synEPOR-edited HSPCs at d14 of erythroid differentiation.

B: Summary of percentage of CD36-APC$^+$ cells across all synEPOR-edited HSPCs at d14 of erythroid differentiation. “+EPO” condition is unedited cells cultured +EPO/-BB; ”-/-” condition is unedited cells cultured -EPO/-BB; all synEPOR-edited conditions were cultured +BB/-EPO. All bars represent mean +/-SEM; all displayed p-values were generated by 2-way ANOVA for multiple comparisons.

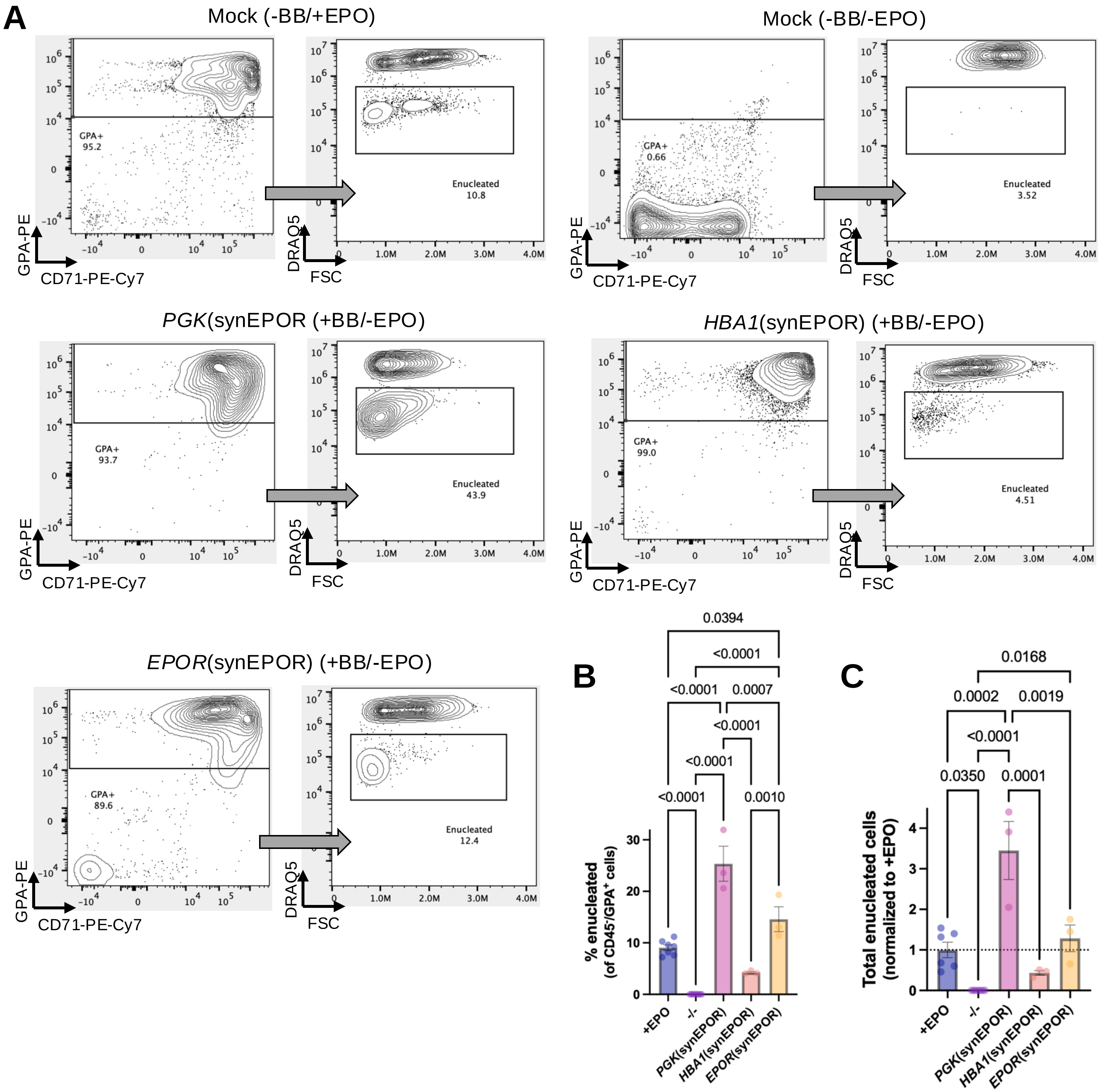

Supplementary Figure 10: Deeper characterization of synEPOR-edited HSPCs using flow cytometry.

A: Representative flow cytometry staining and gating scheme for synEPOR-edited HSPCs at d14 of erythroid differentiation. Arrows indicate that only gated cells are displayed on the subsequent plot.

B: Summary of percentage of enucleated cells across all synEPOR-edited HSPCs at d14 of erythroid differentiation. "+EPO" condition is unedited cells cultured +EPO/-BB; "-/-" condition is unedited cells cultured -EPO/-BB; all synEPOR-edited conditions were cultured +BB/-EPO. All bars represent mean +/-SEM; all displayed p-values were generated by 2-way ANOVA for multiple comparisons.

C: Total CD45-V450^{-}/GPA-PE^{+}/DRAQ5^{-} cell counts at the end of differentiation normalized to +EPO condition. "+EPO" condition is unedited cells cultured +EPO/-BB; "-/-" condition is unedited cells cultured -EPO/-BB; all synEPOR-edited conditions were cultured +BB/-EPO. All bars represent mean +/-SEM; all displayed p-values were generated by 2-way ANOVA for multiple comparisons.

A

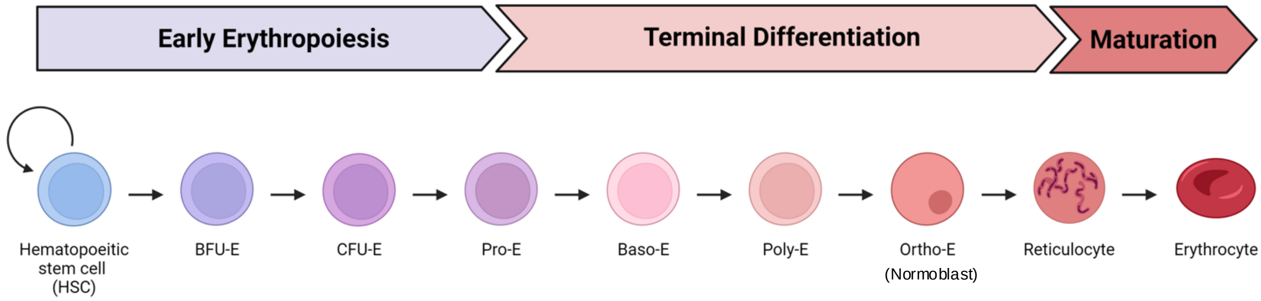

B

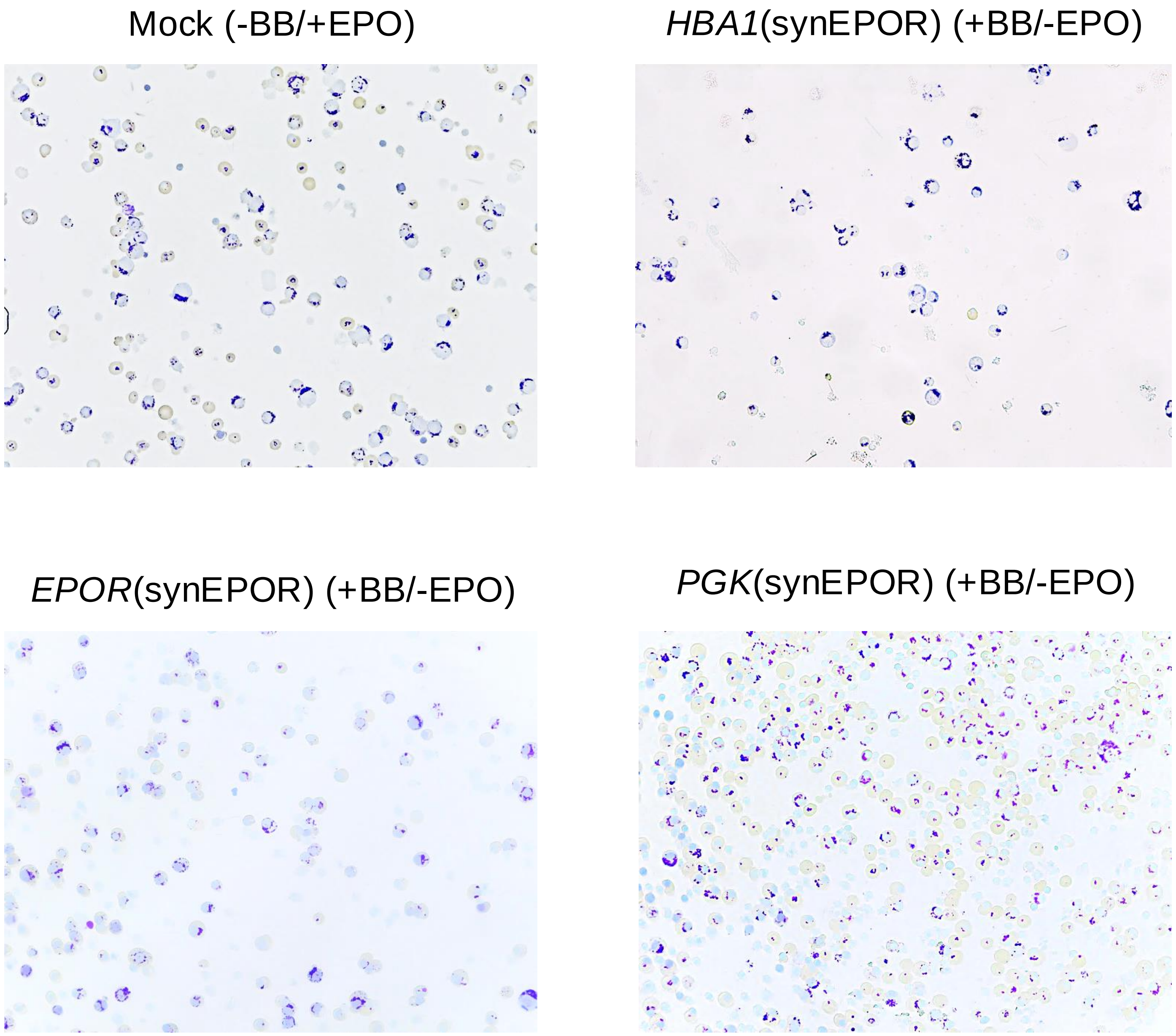

Supplementary Figure 11: Morphological analysis of synEPOR-edited erythroid cells.
A: Diagram representing established stages and cell types over the course of HSC-to-erythrocyte maturation. Created in BioRender. Lesch, B. (2025) https://BioRender.com/j36v879.
B: Representative images of cresyl blue staining (20x magnification) of edited HSPCs at d14 of differentiation.

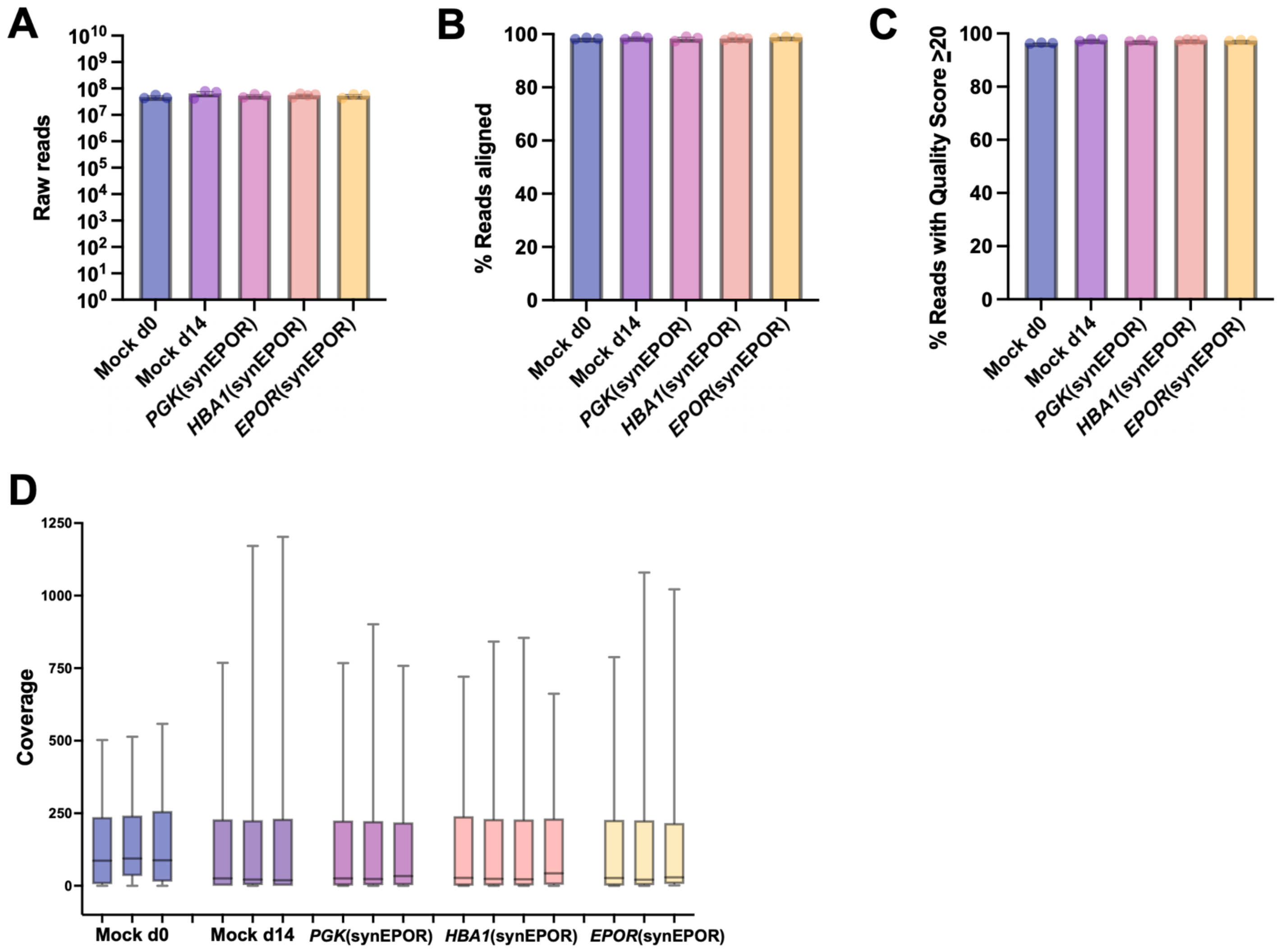

Supplementary Figure 12: RNA-sequencing quality.

A: Summary of reads by condition. All bars represent mean +/-SEM.

B: Percentage of reads aligned to genome by condition. All bars represent mean +/-SEM.

C: Percentage of reads with Quality Score ≥20 by condition. All bars represent mean +/-SEM.

D: Coverage after normalization by condition. Box represents 25th, 50th, and 75th percentiles. Whiskers represent 10th and 90th percentiles.

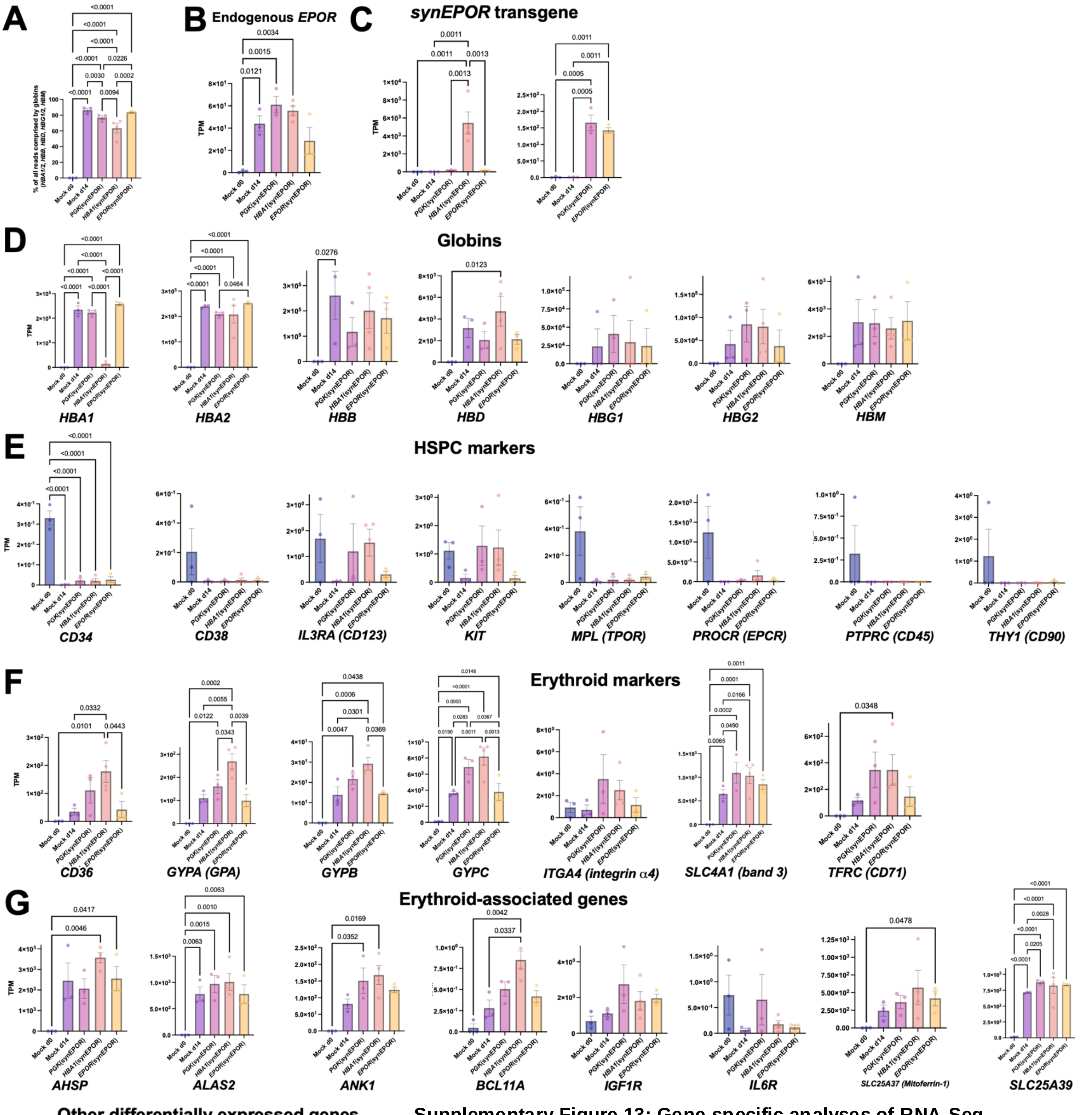

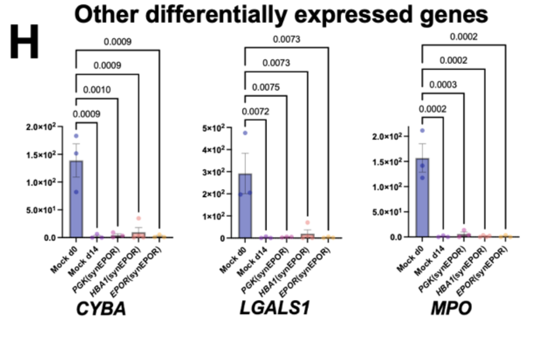

Supplementary Figure 13: Gene-specific analyses of RNA-Seq.
A: Percentage of all reads comprised by globins (includes *HBA1/2*, *HBB*, *HBD*, *HBG1/2*, & *HBM*).
B: Transcripts per million (TPM) of endogenous *EPOR* across conditions.
C: TPM of *synEPOR* transgene across conditions. Right panel has *HBA1*(synEPOR) removed to show differential expression among other conditions.
D: TPM of globin genes across conditions.
E: TPM of HSPC markers across conditions.
F: TPM of erythroid markers across conditions.
G: TPM of erythroid-associated genes across conditions.
H: TPM of other most differentially expressed genes across conditions.
All panels: All bars represent mean +/-SEM; all displayed p-values were generated by 2-way ANOVA for multiple comparisons.

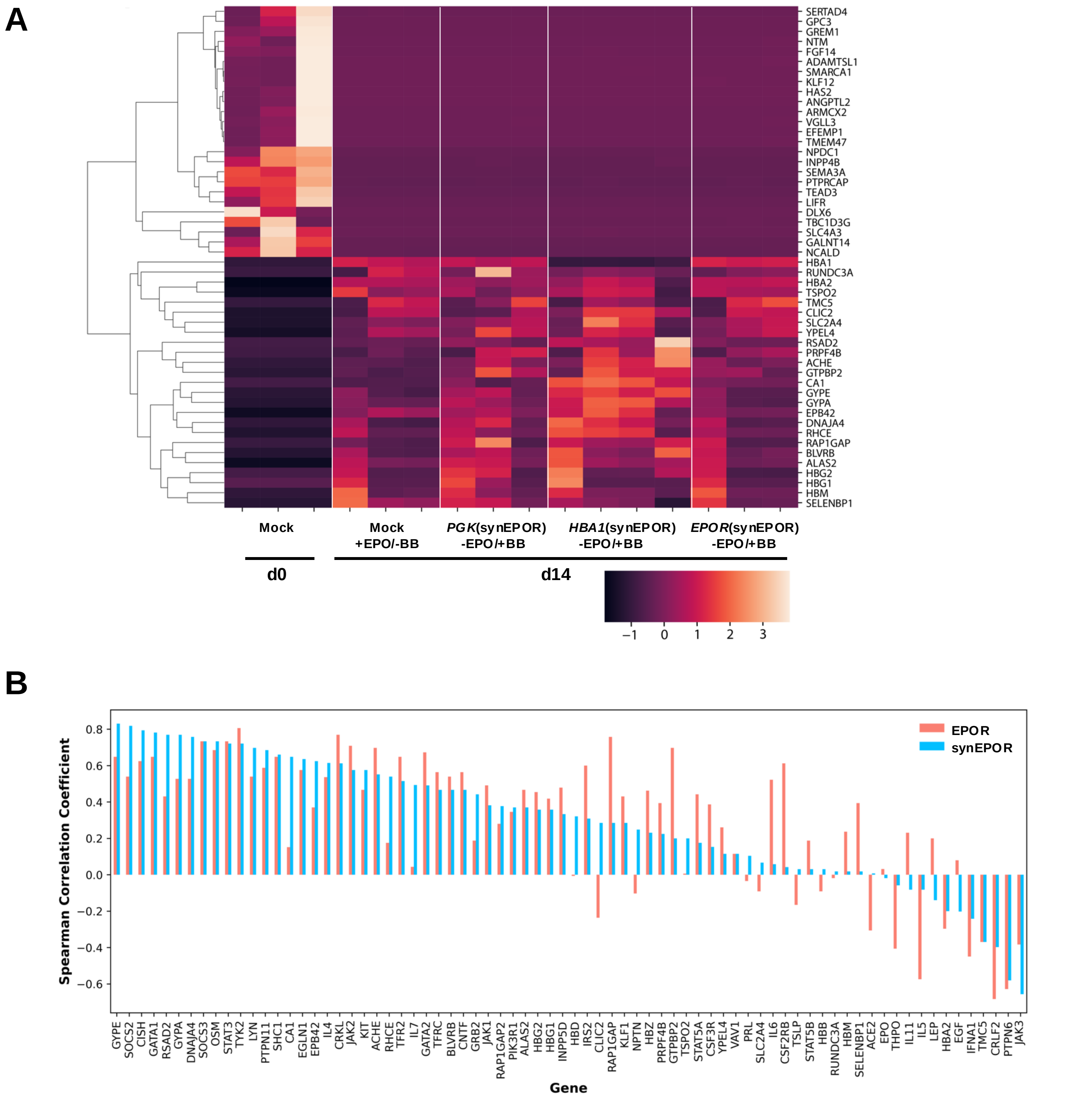

Supplementary Figure 14: Additional analyses of transcriptomic data.

A: Heatmap plotting top 25 differentially upregulated and downregulated genes (comparing d0 vs. all d14 samples).

B: Spearman correlation coefficient is plotted for a specified set of *EPOR*-related genes to depict which genes *synEPOR* adequately mimics in the immediate gene co-expression network of endogenous *EPOR*.

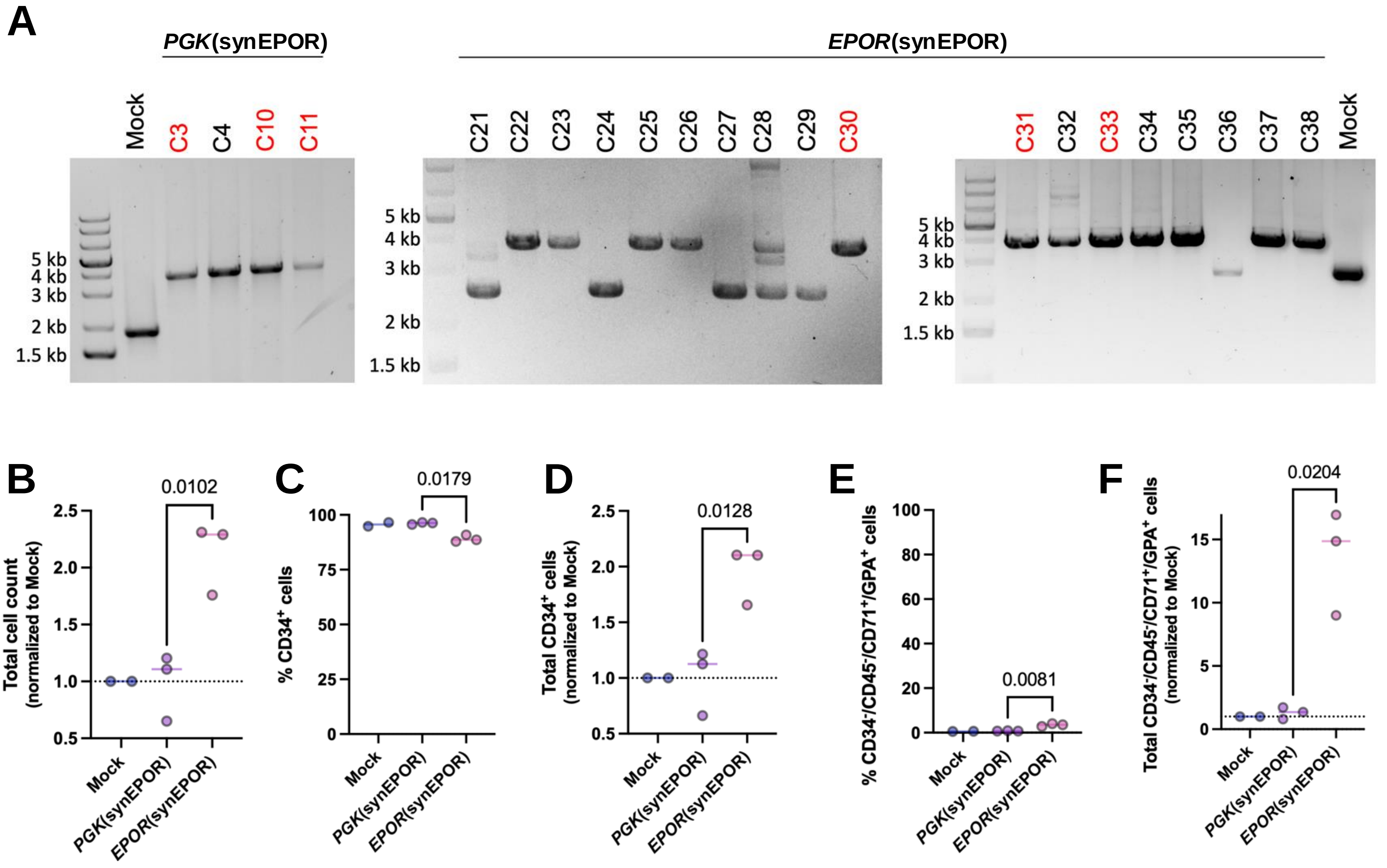

Supplementary Figure 15: Isolation of homozygous synEPOR-edited iPSC clones & downstream HPC differentiation.

A: Agarose gel electrophoresis images of PCR-amplified genomic DNA surrounding the predicted *CCR5* and *EPOR* integration sites which was used to genotype synEPOR-edited iPSC clones. Homozygous clones chosen for downstream analysis are highlighted in red.

B: Total cell counts at the end of iPSC-to-HPC differentiation normalized to unedited cells. All bars represent median; all displayed p-values were generated by 2-way ANOVA for multiple comparisons.

C: Percentage of cells staining CD34-APC$^+$ by flow cytometry following iPSC-to-HPC differentiation. All bars represent median; all displayed p-values were generated by 2-way ANOVA for multiple comparisons.

D: Total CD34-APC$^+$ cell counts at the end of iPSC-to-HPC differentiation normalized to unedited cells. All bars represent median; all displayed p-values were generated by 2-way ANOVA for multiple comparisons.

E: Percentage of cells that acquired erythroid markers (CD34-APC$^-$/CD45-V450$^-$/CD71-PE-Cy7$^+$/GPA-PE$^+$) following iPSC-to-HPC differentiation. All bars represent median; displayed p-value was generated by 2-way ANOVA for multiple comparisons.

F: Total CD34-APC$^-$/CD45-V450$^-$/CD71-PE-Cy7$^+$/GPA-PE$^+$ cell counts at the end of iPSC-to-HPC differentiation normalized to unedited cells. All bars represent median; all displayed p-values were generated by 2-way ANOVA for multiple comparisons.

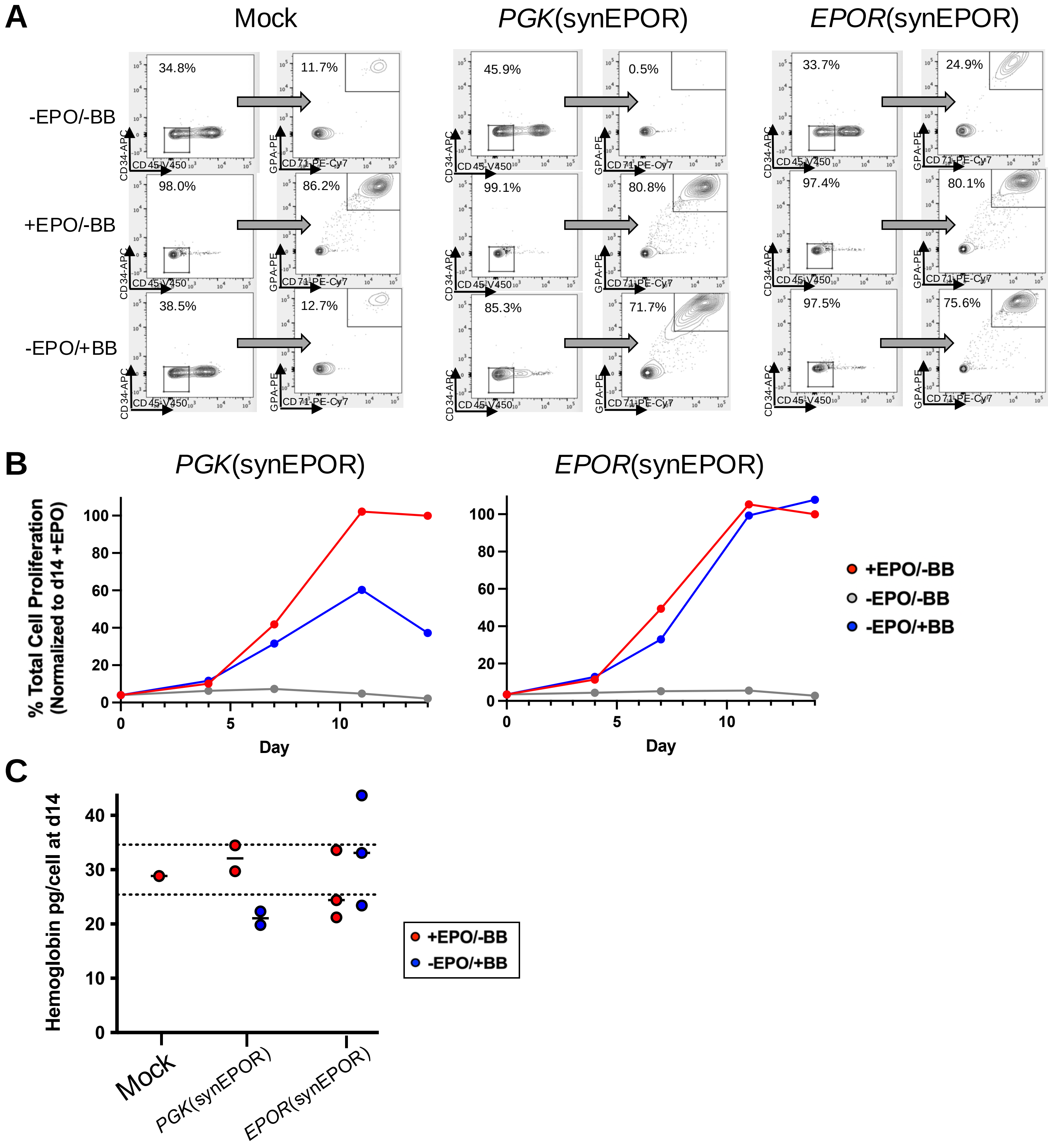

Supplementary Figure 16: Additional analyses of synEPOR-edited iPSC-derived erythroid cells.
A: Representative flow cytometry staining and gating scheme for synEPOR-edited iPSCs at end of erythroid differentiation. Arrows indicate that only gated cells are displayed on the subsequent plot.
B: Percentage of total cell proliferation normalized to clones cultured +EPO over the course of differentiation. Only the clone with greatest proliferation in presence of BB is shown in each editing condition.
C: Hemoglobin production per cell for edited and unedited iPSCs at end of erythroid differentiation. Bars depict median value; dotted lines depict normal range of mean corpuscular hemoglobin concentration in peripheral blood.
