# Supplementary material for: Engineering synthetic signaling receptors to enable erythropoietin-free erythropoiesis
Source: Nat Commun. 2025 Jan 29;16:1140. doi: 10.1038/s41467-025-56239-5 (PMC11779867; doi:10.1038/s41467-025-56239-5)
Supplement: Supplementary file 2 — Description of Additional Supporting Information [file 41467_2025_56239_MOESM2_ESM.docx]

**Supplementary Data File Legends**

**Supplementary Data 1: RNA expression data.**

Transcripts per million (TPM) values for each annotated gene (summed across isoforms) are shown following RNA-Seq in unedited cells at d0 and d14 of erythroid differentiation as well as synEPOR-edited cells at d14 of erythroid differentiation. Mitochondrial and lowly expressed genes were removed (sum NumReads <1). Each replicate within a given condition is displayed as an individual column.

**Supplementary Data 2: Differential gene expression analysis.**

Results from differential gene expression analysis (using DESeq2) are shown following RNA-Seq in unedited cells at d0 and d14 of erythroid differentiation as well as synEPOR-edited cells at d14 of erythroid differentiation. Each sheet shows a different comparative analysis. “baseMean” reports the average of the normalized count values for a gene across all samples, calculated after dividing by size factors. “log2FoldChange” reports the logarithm (base 2) of the fold change between the two samples being compared. “p-value” reports the raw p-value calculated for each gene during differential expression analysis, without any correction for multiple comparisons. “padj” refers to the p-value following adjustment to account for multiple comparisons by Wald test. “minus_log10p” refers to the negative logarithm (base 10) of the adjusted p-value. Cells with NA indicate genes for which the the fold change and/or p-value was unable to be calculated due to low expression and/or an extreme count outlier.

**Supplementary Data 3: Gene ontology enrichment analysis.**

Results from gene ontology (GO) enrichment analysis (using Enrichr) are shown following RNA-Seq in unedited cells at d0 and d14 of erythroid differentiation as well as synEPOR-edited cells at d14 of erythroid differentiation. The top 50 up- and down-regulated genes for a given comparison were used as input; each sheet shows the output of different given comparisons. “P-value” refers to the raw p-value calculated for each gene during differential expression analysis, without any correction for multiple comparisons. “Adjusted P-value” reports the p-value following Benjamini-Hochberg False Discovery Rate adjustment to account for multiple comparisons. “Odds Ratio” was calculated by comparing the proportion of genes belonging to a specific GO term within the set of differentially expressed genes vs. the proportion of genes belonging to that same GO term in the background set of all genes. “Combined Score” was calculated by multiplying the natural logarithm of the p-value by the z-score, where the z-score represents the deviation of a term's rank from its expected rank. “Genes” refers to the given genes that were significantly enriched within a specific GO term.
