# Supplementary material for: Engineering synthetic signaling receptors to enable erythropoietin-free erythropoiesis
Source: Nat Commun. 2025 Jan 29;16:1140. doi: 10.1038/s41467-025-56239-5 (PMC11779867; doi:10.1038/s41467-025-56239-5)
Supplement: Supplementary file 6 — Reporting Summary [file 41467_2025_56239_MOESM6_ESM.pdf]

nature portfolio

Corresponding author(s): Kyle Cromer

Last updated by author(s): Jan 8, 2025

Reporting Summary

Nature Portfolio wishes to improve the reproducibility of the work that we publish. This form provides structure for consistency and transparency in reporting. For further information on Nature Portfolio policies, see our Editorial Policies and the Editorial Policy Checklist.

Statistics

For all statistical analyses, confirm that the following items are present in the figure legend, table legend, main text, or Methods section.

n/a	Confirmed	
☐	☒	The exact sample size (*n*) for each experimental group/condition, given as a discrete number and unit of measurement
☐	☒	A statement on whether measurements were taken from distinct samples or whether the same sample was measured repeatedly
☐	☒	The statistical test(s) used AND whether they are one- or two-sided<br>*Only common tests should be described solely by name; describe more complex techniques in the Methods section.*
☐	☒	A description of all covariates tested
☒	☐	A description of any assumptions or corrections, such as tests of normality and adjustment for multiple comparisons
☐	☒	A full description of the statistical parameters including central tendency (e.g. means) or other basic estimates (e.g. regression coefficient) AND variation (e.g. standard deviation) or associated estimates of uncertainty (e.g. confidence intervals)
☐	☒	For null hypothesis testing, the test statistic (e.g. *F*, *t*, *r*) with confidence intervals, effect sizes, degrees of freedom and *P* value noted<br>*Give P values as exact values whenever suitable.*
☒	☐	For Bayesian analysis, information on the choice of priors and Markov chain Monte Carlo settings
☒	☐	For hierarchical and complex designs, identification of the appropriate level for tests and full reporting of outcomes
☐	☒	Estimates of effect sizes (e.g. Cohen's *d*, Pearson's *r*), indicating how they were calculated

Our web collection on statistics for biologists contains articles on many of the points above.

Software and code

Policy information about availability of computer code

Data collection	FACS Diva was used to collect flow cytometry data.<br>QuantaSoft v1.7 was used to measure ddPCR-based vector genomes.<br>TotalChrom was used to quantify the curve of the peaks for hemoglobin tetramer HPLC.
Data analysis	GraphPad Prism v9 was used to plot data and conduct statistical analyses.<br>Visualization: Adobe Illustrator, BioRender, ChimeraX,<br><br>RNA-Seq Alignment: Salmon (1.10.2), bowtie2 (2.5.2)<br>LS Method: https://github.com/bdhammel/least-squares-ellipse-fitting<br>Folding Predictions: AlphaFold (2.3.2)<br><br>Python (3.11.8) packages:<br>pandas (2.1.1), numpy (1.26.0), matplotlib (3.8.0), seaborn (0.12.2), scipy (1.11.3), sklearn (scikit-learn) (1.3.1), BioPython (1.81), networkx (3.1), subprocess, collections, os, json

For manuscripts utilizing custom algorithms or software that are central to the research but not yet described in published literature, software must be made available to editors and reviewers. We strongly encourage code deposition in a community repository (e.g. GitHub). See the Nature Portfolio guidelines for submitting code & software for further information.

Data

Policy information about availability of data

All manuscripts must include a data availability statement. This statement should provide the following information, where applicable:

- Accession codes, unique identifiers, or web links for publicly available datasets
- A description of any restrictions on data availability
- For clinical datasets or third party data, please ensure that the statement adheres to our policy

RNA-seq data have been deposited in the NCBI Gene Expression Omnibus database (accession no. GSE285656) and are publicly available as of the date of publication. Sequencing reads were aligned to the GRCh38 reference human genome (NCBI Sequence Read Archive database; accession no. PRJNA31257). The data for all figures in this study are provided in the Source Data file.

Research involving human participants, their data, or biological material

Policy information about studies with human participants or human data. See also policy information about sex, gender (identity/presentation), and sexual orientation and race, ethnicity and racism.

Reporting on sex and gender	HSPCs were isolated from both male and female donors.
Reporting on race, ethnicity, or other socially relevant groupings	No exclusion criteria applied regarding ethnicity, race, or other socially relevant groupings.
Population characteristics	Human-derived samples were de-identified prior to use in our studies and we therefore had no access to characteristics of patient samples used in this study.
Recruitment	Participants were recruited by Stanford's Binns Family Cord Blood Program. Selection bias in this case would result from the population of patients who have access to care at Stanford Hospital. However, we believe that selection bias is accounted for due to the fact that each patient-derived sample was subdivided and then subjected to various treatment conditions, thereby accounting for any donor-to-donor variability inherent to the samples.
Ethics oversight	Informed patient consent was acquired and patients were recruited in accordance with IRB protocol number 33813.

Note that full information on the approval of the study protocol must also be provided in the manuscript.

Field-specific reporting

Please select the one below that is the best fit for your research. If you are not sure, read the appropriate sections before making your selection.

[x] Life sciences [ ] Behavioural & social sciences [ ] Ecological, evolutionary & environmental sciences

For a reference copy of the document with all sections, see nature.com/documents/nr-reporting-summary-flat.pdf

Life sciences study design

All studies must disclose on these points even when the disclosure is negative.

Sample size	Sample sizes used in this study were determined by conducting power analyses given the degree of variation observed in previous Cas9/AAV6-mediated genome editing studies (Dever et al. Nature 2016; Pavel-Dinu et al. Nat Commun 2019; Gomez-Ospina et al. Nat Commun 2019).
Data exclusions	No data was excluded from the analyses.
Replication	Experiments were performed via multiple donor samples on different days with the appropriate positive and negative controls. All data was reproducible across experiments.
Randomization	Not relevant to the study. Treatment groups all originated from the same pool of initial cells.
Blinding	Blinding was not possible. Blinding not relevant as no investigator-based data. All data reported was quantified via instruments and analyzed together across treatment conditions.

Reporting for specific materials, systems and methods

We require information from authors about some types of materials, experimental systems and methods used in many studies. Here, indicate whether each material, system or method listed is relevant to your study. If you are not sure if a list item applies to your research, read the appropriate section before selecting a response.

Materials & experimental systems

n/a	Involved in the study
☐	☒ Antibodies
☐	☒ Eukaryotic cell lines
☒	☐ Palaeontology and archaeology
☒	☐ Animals and other organisms
☒	☐ Clinical data
☒	☐ Dual use research of concern
☒	☐ Plants

Methods

n/a	Involved in the study
☒	☐ ChIP-seq
☐	☒ Flow cytometry
☒	☐ MRI-based neuroimaging

Antibodies

Antibodies used: CD34 APC (1:50 dilution; 561; BioLegend, San Diego, CA, USA), CD45 V450 (1:50 dilution; 2µL in 100µl of pelleted RBCs in 1×PBS buffer; HI30; BD Biosciences), CD36 PE (1:50 dilution; 5-271; BioLegend), CD71 PE-Cy7 (1:500 dilution; OKT9; Affymetrix, Santa Clara, CA, USA), and CD235a PE (GPA) (1:500 dilution; GA-R2; BD Biosciences) or GPA PE-Cy5 (1:500 dilution; GA-R2; BD Biosciences)

Validation: Prior studies in the lab had thoroughly validated all antibodies used in this study (Cromer et al. Nat Medicine 2021; Luna et al Nat Biomed Eng 2024).

Eukaryotic cell lines

Policy information about cell lines and Sex and Gender in Research

Cell line source(s): HEK293T cells (CRL-1573, ATCC, Manassas, VA, USA)

Authentication: None of the cell lines used were authenticated.

Mycoplasma contamination: Cell lines were not tested for mycoplasma contamination.

Commonly misidentified lines (See ICLAC register): No commonly misidentified cell lines were used in the study.

Plants

Seed stocks: *Report on the source of all seed stocks or other plant material used. If applicable, state the seed stock centre and catalogue number. If plant specimens were collected from the field, describe the collection location, date and sampling procedures.*

Novel plant genotypes: *Describe the methods by which all novel plant genotypes were produced. This includes those generated by transgenic approaches, gene editing, chemical/radiation-based mutagenesis and hybridization. For transgenic lines, describe the transformation method, the number of independent lines analyzed and the generation upon which experiments were performed. For gene-edited lines, describe the editor used, the endogenous sequence targeted for editing, the targeting guide RNA sequence (if applicable) and how the editor was applied.*

Authentication: *Describe any authentication procedures for each seed stock used or novel genotype generated. Describe any experiments used to assess the effect of a mutation and, where applicable, how potential secondary effects (e.g. second site T-DNA insertions, mosiacism, off-target gene editing) were examined.*

Flow Cytometry

Plots

Confirm that:

☐ The axis labels state the marker and fluorochrome used (e.g. CD4-FITC).

☒ The axis scales are clearly visible. Include numbers along axes only for bottom left plot of group (a 'group' is an analysis of identical markers).

☒ All plots are contour plots with outliers or pseudocolor plots.

☒ A numerical value for number of cells or percentage (with statistics) is provided.

Methodology

Sample preparation: Samples were stained with fluorochrome-conjugated antibodies against surface markers of interest. Staining was performed at 4C, in the dark, and cells suspended in PBS.

Instrument: FACS Aria II

Software	FACS Diva was used to collect data. FlowJo v10 was used to analyze data.
Cell population abundance	N/A
Gating strategy	Gating strategy employed to characterize HSPC-to-erythroid differentiation can be found in Supplementary Figure 1.

☒ Tick this box to confirm that a figure exemplifying the gating strategy is provided in the Supplementary Information.
